# CellPAINT: Turnkey Illustration of Molecular Cell Biology

**DOI:** 10.3389/fbinf.2021.660936

**Published:** 2021-03-29

**Authors:** Adam Gardner, Ludovic Autin, Daniel Fuentes, Martina Maritan, Benjamin A. Barad, Michaela Medina, Arthur J. Olson, Danielle A. Grotjahn, David S. Goodsell

**Affiliations:** ^1^Department of Integrative Structural and Computational Biology, The Scripps Research Institute, La Jolla, CA, United States; ^2^Research Collaboratory for Structural Bioinformatics Protein Data Bank, Rutgers, The State University of New Jersey, Piscataway, NJ, United States

**Keywords:** molecular illustration, cellular structure, cryo-electron tomography, biomolecular assembly, computational biology

## Abstract

CellPAINT is an interactive digital tool that allows non-expert users to create illustrations of the molecular structure of cells and viruses. We present a new release with several key enhancements, including the ability to generate custom ingredients from structure information in the Protein Data Bank, and interaction, grouping, and locking functions that streamline the creation of assemblies and illustration of large, complex scenes. An example of CellPAINT as a tool for hypothesis generation in the interpretation of cryoelectron tomograms is presented. CellPAINT is freely available at http://ccsb.scripps.edu/cellpaint.

## Introduction

We're in the middle of a revolution in biology, as molecular biology rapidly merges with cell biology. New advances in electron microscopy are providing increasingly detailed views of the molecular structure of large cellular assemblies (such as the nuclear pore) and the *in situ* structure of these assemblies within cells (Beck and Baumeister, 2016; Irobalieva et al., 2016). Large gaps still remain, however, in what can be directly observed. Currently, we still need to fill these gaps using integrative modeling (Rout and Sali, 2019; Ziegler et al., 2021), with the goal of gathering the current state of knowledge and building models that are consistent with what is known.

**Figure 1:**
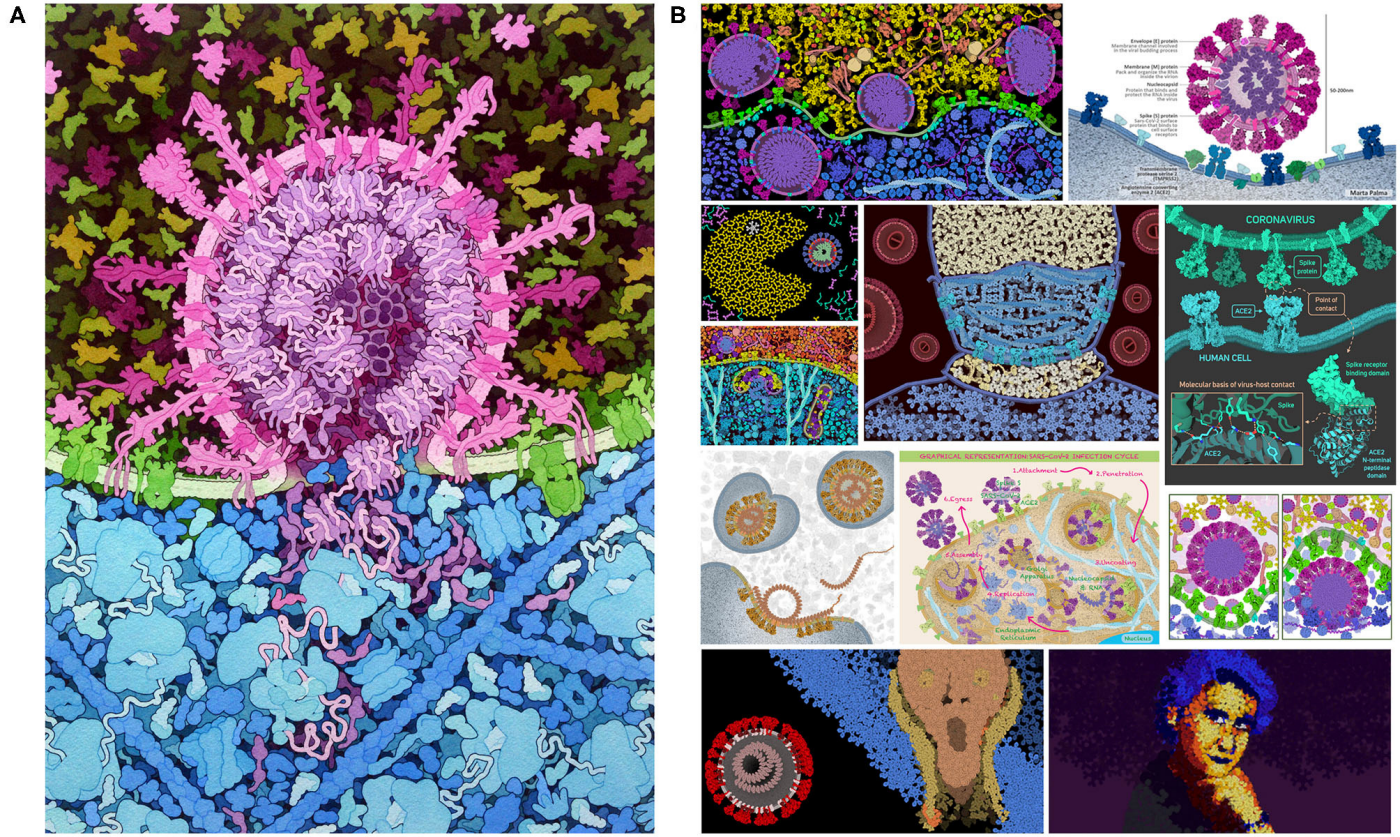
Coronavirus illustration. **(A)** Integrative illustration of SARS-CoV-2 (magenta) fusing with an endosomal membrane (green) and releasing its genomic RNA (purple) into the cytoplasm (blue), created with traditional painting techniques. **(B)** Selected entries to the 2020 CellPAINT Coronavirus Contest at the RCSB Protein Data Bank.

For the past 30 years, we have used an integrative approach to create illustrations of portions of living cells with molecular detail (Goodsell, 1991, 2009; Goodsell et al., 2020a). The goal of these illustrations is to include all macromolecules at the proper size, concentration, and location, and representing any interactions that may occur (see Figure 1A for a recent example). An enormous amount of information is available through resources, such as the Protein Data Bank (Berman et al., 2000), UniProt (UniProt Consortium, 2019), and PubMed (NCBI Resource Coordinators, 2018), but inevitably, there are aspects of these illustrations that still require artistic license to incorporate speculation and hypotheses about aspects that are still under study. These illustrations are work-intensive, building on decades of experience both in the gathering of appropriate data and in the rendering of the final image (Goodsell, 2016).

We initiated the CellPAINT project in 2016 to allow students, educators, and researchers to create these types of integrative illustrations (Gardner et al., 2018). The program is designed much like traditional digital painting software, with a canvas and molecular “brushes” for building a cellular scene from its component proteins, membranes, DNA, and other molecules. CellPAINT manages the details of scale and interaction, allowing users to focus on the biology of the scene and explore how arrangements of molecules can lead to emergent physiological features.

When first seeing CellPAINT, users often compare it to BioRender (https://Biorender.com). BioRender is a highly-developed turnkey tool for creating the biological schematics that are widely used in journal articles and professional presentations. As with CellPAINT, BioRender provides a selection of sprites that may be interactively added to a scene. The goals of CellPAINT, however, are quite different from BioRender, and thus CellPAINT is built with a different set of underlying constraints and assumptions. CellPAINT seeks to generate a scene that reflects the physical size and properties of the molecules, allowing accurate illustration of a particular portion of a cell or virus. BioRender, on the other hand, is highly effective for presenting higher-level concepts, and icons representing atoms, molecules, cells, and even entire organisms can be easily combined into a single figure, along with labels and other graphical elements.

Previous versions of CellPAINT have been primarily deployed in educational settings. For example, we mounted a visualization contest at PDB-101, the educational portal of the RCSB Protein Data Bank (Berman et al., 2000). The contest was open to people of all ages and solicited entries in two categories: scientific art designed to present the subject with scientific accuracy, and fine art limited only by the artist's imagination. As shown in Figure 1B, the response showed a remarkable range of creativity in the entries and served to underscore the flexibility of CellPAINT to enable a wide range of imagery (for more information, see the entry for 06/07/2020 at http://pdb101.rcsb.org/news/2020). The new version of CellPAINT presented here, CellPAINT-2.0, adds many additional capabilities and paves the way for use of interactive integrative illustration as a tool for hypothesis generation in research, as a gateway for more quantitative approaches to modeling the molecular structure of living cells. We present a test case of using CellPAINT for interpretation of cryo-focused ion beam (FIB)-milled tomograms of mitochondria functioning within their native, cellular environment. Tomograms of cellular landscapes are challenging to interpret, as they are often crowded with tightly packed subcellular organelles and a diverse array of protein complexes. As demonstrated below, CellPAINT proved to be an effective tool for interactive interpretation of these complex experimental images.

## Methods

### Overview of CellPAINT

**Figure 2:**
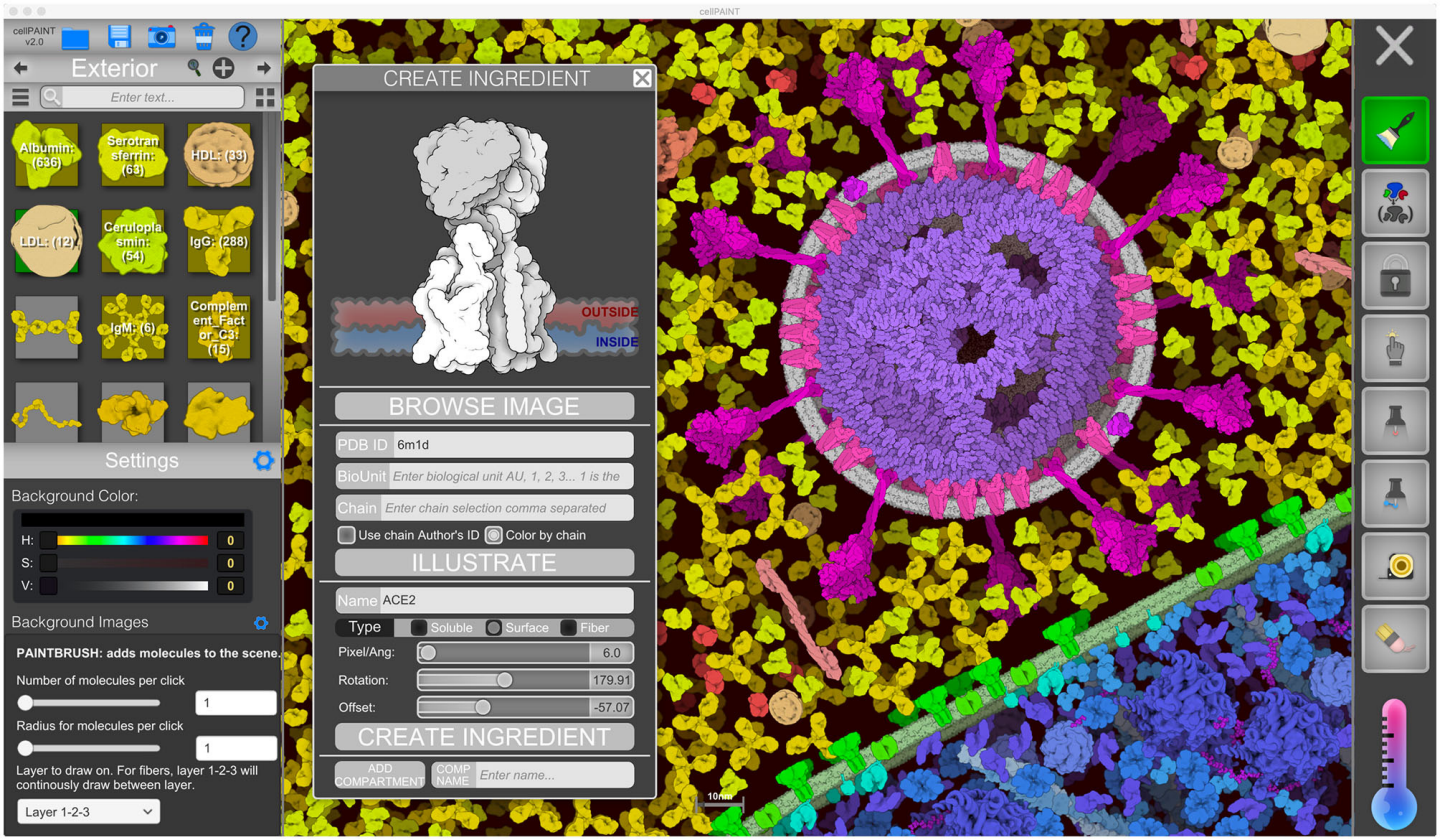
CellPAINT user interface. Here, the user is illustrating a scene using the default ingredients for a simple eukaryotic cell, blood plasma, and coronavirus. A new ingredient is being created for ACE2 based on atomic coordinates fetched from the Protein Data Bank.

CellPAINT is built in Unity, which provides much of the infrastructure for generating the interface, managing user interaction, and controlling the physics of the scene (Figure 2). CellPAINT relies on an overarching data structure called a *recipe*, as originally defined in CellPACK (Johnson et al., 2015). In the recipe, cells and organelles are defined as *compartments* and molecules within are defined as *ingredients*. In CellPACK, scenes are automatically built in 3D and ingredients are represented by 3D meshes or grapes or beads. In contrast, CellPAINT uses a 2.5D paradigm for manually building the scene: ingredients are provided as 2D sprites that are allowed to rotate only in the plane of the screen, and scenes are constructed of a foreground layer and two background layers progressively depth cued to the color of the background. The 2.5D paradigm has advantages and disadvantages, as described below in the Discussion.

The user interface of CellPAINT is shown in Figure 2. A control panel on the left includes options for managing files and opening various pop-up windows for ingredient management, such as the “Create Ingredient” panel shown in the figure. Palettes of ingredients for each of the compartments allow users to select molecules to brush into the scene. Various advanced options are included at the bottom of the window to tune the behaviors of the different painting options. Buttons in the right panel control the painting options. These include methods to add and erase ingredients in the scene, to pin them in place or to each other, to group or lock individual ingredients or collections of them, and to make measurements between ingredients. Several options in the new release are described in more detail below. Full documentation and tutorials are available at the CellPAINT site at http://ccsb.scripps.edu/cellpaint.

### CellPAINT Design, Development, and Testing

Several overarching goals have driven our design for CellPAINT. First, CellPAINT is intended to be a turnkey method that does not require deep technical knowledge of structural biology or molecular graphics. Second, CellPAINT is designed to streamline incorporation of appropriate scale relationships and the hierarchical structure of cellular environments as users create images, through tight association with experimental structural data. As discussed throughout this report, much of the design challenge has been in balancing these two goals, in order to create a tool that exposes an appropriate number of user-tunable parameters to capture the relevant biology while not overwhelming non-expert users or detracting from the interactive experience.

CellPAINT is implemented within the Unity development environment, streamlining the addition of new features and deployment on a wide variety of platforms. This has allowed a nimble development cycle, with multiple releases incorporating and tuning new features. User feedback is obtained at multiple levels. A core set of “power users” in research and in education provide rapid feedback on new features. With major releases, user feedback is solicited from the wider research and education community. This has included informal release and solicitation of feedback on our website, SourceForge pages, and social media, and targeted testing in classroom settings and in contests with the RCSB Protein Data Bank. In the classroom activities and contests, we provide a questionnaire with specific questions about usability of new features and open-ended questions on feature requests and the types of systems that are of interest. Some of the insights gained from this design and feedback loop are described in Results and Discussion.

### CellPAINT User Interface

Managing recipes of increasing complexity and dealing with expanded tools with multiple options necessitated a complete redesign of the original CellPAINT user interface. To avoid confusion when painting we opted for a dedicated painting area free from overlaid controls. To facilitate a clean and intuitive interface, we hide options/features that aren't commonly accessed in pop ups or toggles. For example, we automatically display options for each tool in the lower left panel only when the tool is active. Managing and searching through recipe compartments with many ingredients was particularly cumbersome in the original CellPAINT. Originally, we designed hexagonal tiling of ingredients as a digital facsimile of a painter's palette, where an image of the ingredient represents a dab of paint. However, as the image of the ingredient was the only visual cue, it quickly became difficult to parse large recipes to find specific ingredients. As we scaled up for more complex paintings, the inadequacies of this approach became more apparent. To help solve these issues we implemented a simple string search query so the user can quickly find ingredients in the recipe by name. We also implemented a switch from the grid view to a list view that includes a single column with exposed ingredient titles and a small thumbnail of the sprite.

### Creating Custom Ingredients

Users of previous versions of CellPAINT have uniformly made one common feature request: the ability to import their own molecules into the software. This seemingly simple request posed many challenges, including the heterogeneity and diversity of coordinate files deposited in the Protein Data Bank; the need to specify parameters for membrane interaction or fiber generation; and the management of scale, viewpoints, colors, and all the other things that users would want to customize. In the “Create Ingredient” window, we have implemented a basic toolbox for creating biomolecular sprites, that necessarily incorporates a number of compromises to simplify the process and avoid implementing a full 3D molecular viewer within CellPAINT:

- Basic relevant information is queried from the RCSB-PDB REST-API (https://data.rcsb.org/#rest-api) and the PDBe REST-API (https://www.ebi.ac.uk/pdbe/api/doc/), and basic options are exposed to the user for choosing chains or biological units;
- PDB ID and basic options are sent to our server that gathers atomic coordinates and calculates the longest axis in the structure. The viewpoint is chosen automatically based on the longest axis in the structure and files are prepared and saved for the rendering;
- Rendering of cartoony sprites is done with Illustrate (Goodsell et al., 2019) using a coarse representation that captures the overall size and shape of the molecule;
- users choose between treating the molecule as soluble, membrane-bound, or part of a fiber and use simple sliders to define their behaviors.

These simple options provide rapid, turnkey creation of new ingredients, but also impose limits on the final sprites. For users who want finer control, the “Create Ingredient” tool also allows input of any 2D image with a transparent background, which can be used to build soluble, membrane-bound, or fiber sprites.

### Ingredient Representation and Colliders

As described in the original version, collisions, constraints, and diffusion rely on the Box2D physics engine provided in Unity. Every ingredient is defined as a rigid body associated with one or more collider proxies depending on its type (soluble, membrane-bound, or fiber). Every rigid body is associated with a physics layer tag that allows filtering of collision queries using a Layer Collision Matrix. This allows three visual layers, where ingredients in the front layer don't collide with other layers. Several collision pairs are exposed to the user and may be turned on or off: Protein-Protein, Fiber-Protein, Fiber-Fiber, and Membrane-Membrane collisions.

**Figure 3:**
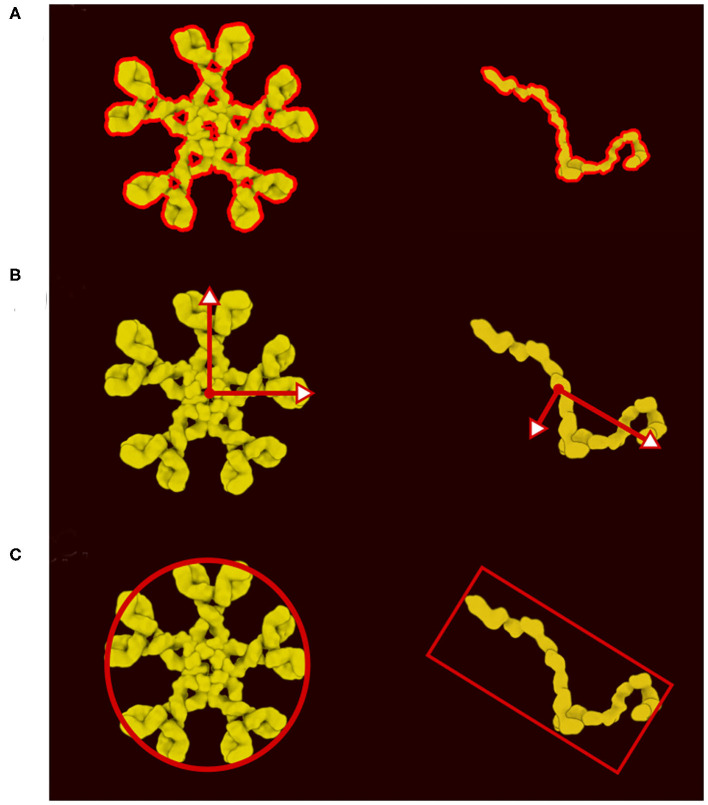
Definition of colliders. **(A)** 2D polygon collider defined from the image contour. **(B)** Eigenvector and eigenvalue calculated from the polygon vertices. **(C)** Main proxy collider chosen from the eigenvector.

In the new version of CellPAINT, we have further developed and automated the definition of colliders for all ingredient types. Every ingredient has one main collider that will best represent its shape. The ideal collider would be a detailed 2D polygon that follows the contour of the molecule, as shown in Figure 3A. However, evaluating collisions with the 2D polygon is currently a bottleneck in the simulation performance, so we use a more approximate shape to reach the largest number of molecules on screen. We currently choose a single primitive shape (circle or rectangle) per molecule, but in future versions, as hardware performance continues to improve, a combination of simple shapes is a logical next step. To automatically find this minimal shape, we determine the eigen decomposition of the covariance matrix of the vertices of the contour (Figure 3B). If the difference between the two eigenvalues is less than a threshold (1.15) a circle collider will be used, otherwise a rectangular collider is used. The size and center of the collider is based on the eigen decomposition, using the first value (X-axis) for the circular collider and the eigenvalues for the rectangular collider (Figure 3C).

#### Representation of Membranes and Fibers

Membrane-bound and fiber sprites also include higher-order behaviors when painted into scenes and require a more complex collider representation. Fibers (like DNA or RNA) are generated as an articulated chain of subunits. By default, when long fibers are drawn, they begin in the foreground layer and then jump periodically to other layers, giving an impression that they fill the available depth of the image. Options are also available to enforce placement of fibers entirely in the foreground or in one of the two background layers.

**Figure 4:**
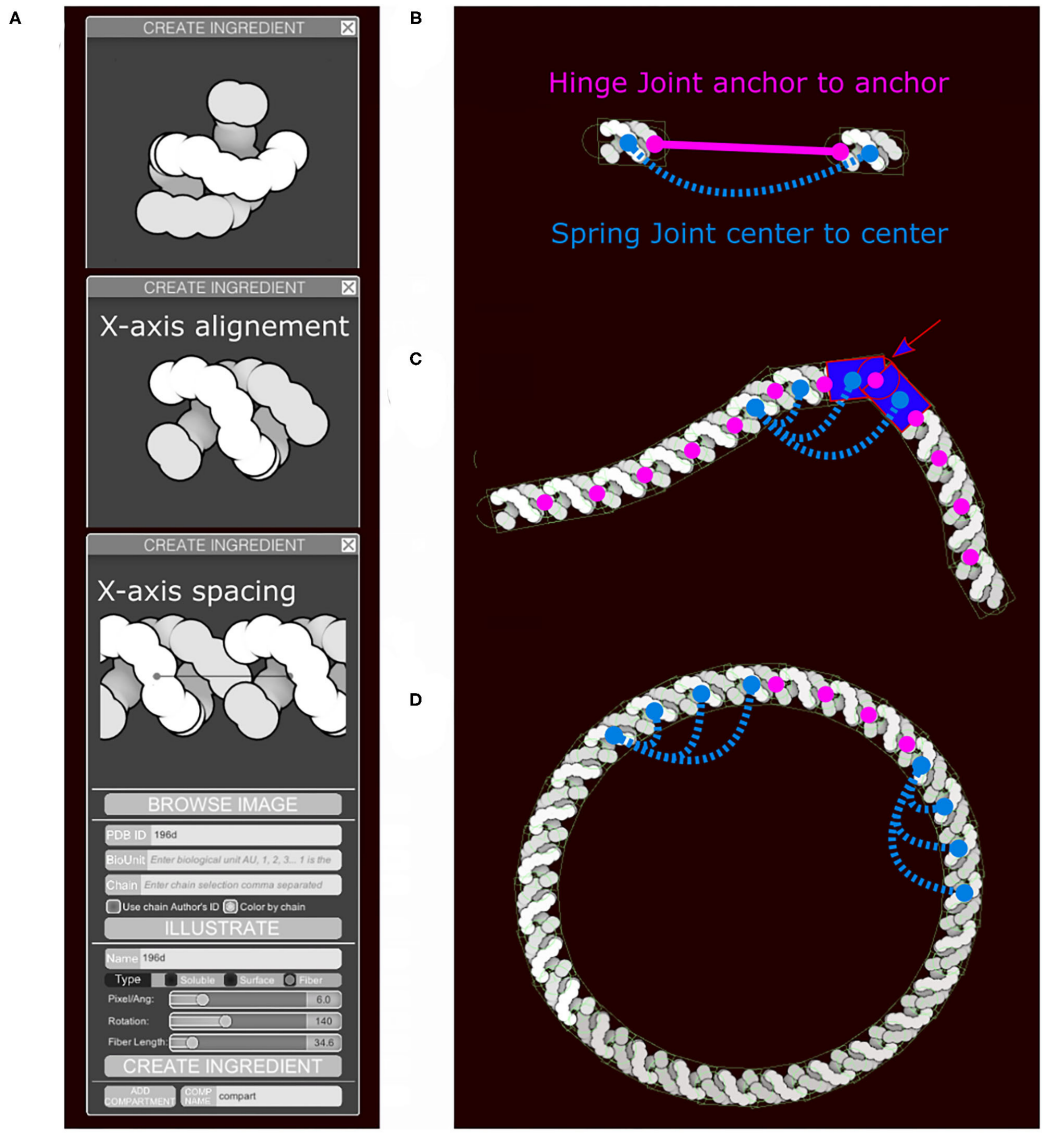
Fiber generation and constraints. DNA structure from PDB ID 196D is used as an example. **(A)** Subunit is aligned by the user along the horizontal axis and a repetition/spacing distance is defined, using sliders in the user interface. **(B)** Two subunits are separated to illustrate the colliders and the joints in use. A rectangular main collider defines the steric properties of each subunit and is the anchor for the persistence length spring joint (blue). The two circular colliders at each end of the subunit serve as anchors for the hinge joint in magenta and do not see the colliders on the neighboring subunit. The hinge line length (magenta) will be constrained to be zero, the spring line length (blue) is always proportional to the spacing times N, the neighbors index defining the number of segments. **(C)** The DNA fiber with proper spacing, illustrating the second role of the circle collider (arrow) that fills the gap for acute angle between subunit. **(D)** Circular DNA showing the role of the spring joints in maintaining a persistence length.

Sliders in the “Create Ingredient” tool allow users to define the spacing of the subunits and their relative rotation (Figure 4A). In addition to the main collider, two additional circle colliders are positioned around the center at a distance corresponding to the spacing value specified. The two circles serve two purposes (Figures 4B,C). First, they act as anchor points for the hinge joint. A hinge joint, as defined in Unity, allows a rigid body to be attached to a point in space or in another rigid body around which it can rotate. The rotation happens in response to a collision. Optionally, angle limits can be applied to limit the rotation. Second, they act as steric colliders to fill the gap when the two subunits are at an acute angle. Circle colliders radii and rectangular collider height use the smallest eigenvalue (Y-axis). The rectangular collider width uses the largest eigenvalue (X-axis).

Persistence length is controlled using additional spring joints that connect non-consecutive subunits at their center. A spring joint allows two rigid bodies to be attached together as if by a spring. The spring will apply a force along its axis between the two objects, attempting to keep them a given distance apart (Figure 4D). The number of additional springs depends on the molecule type. Several default fiber types that are distributed with CellPAINT have been tuned to match biological properties: DNA is very stiff and uses 10 springs, RNA is highly flexible and uses no additional springs, and membranes use an intermediate number of three springs. This parameter is not currently exposed to the user, so we use the default value of three springs for all user-defined fibers. In future work, we will explore methods to provide persistence length as a tunable parameter, as well as exploring methods to model higher-order geometries, such as helices. These types of refinements will be particularly necessary as we move to 3D versions of CellPAINT, as described in the Discussion.

Membranes are treated much like fibers, with rectangular colliders over each subunit and circular colliders at the ends, forming the joint between segments. Additional small colliders are added above and below the membrane to enforce a minimum distance between membranes. As users draw membranes, two behaviors are implemented. If the beginning and end are drawn within a segment length, the membrane is closed and a textured background mesh is built within the resultant closed compartment. The background mesh is computed based on a 2D triangulation using the segment position (https://www.flipcode.com/archives/Efficient_Polygon_Triangulation.shtml) and stays dynamic. Closed vesicles should be drawn in clockwise direction to enforce the same directionality as is used for the membrane-bound ingredients. As users draw membranes, a small icon is displayed to cue them on this directionality of drawing. If the membrane is not closed, the ends are pinned in place by default, allowing the user to place segments of membrane (such as cell surfaces or segments of large organelles) in a desired location.

#### Representation of Membrane-Bound Proteins

**Figure 5:**
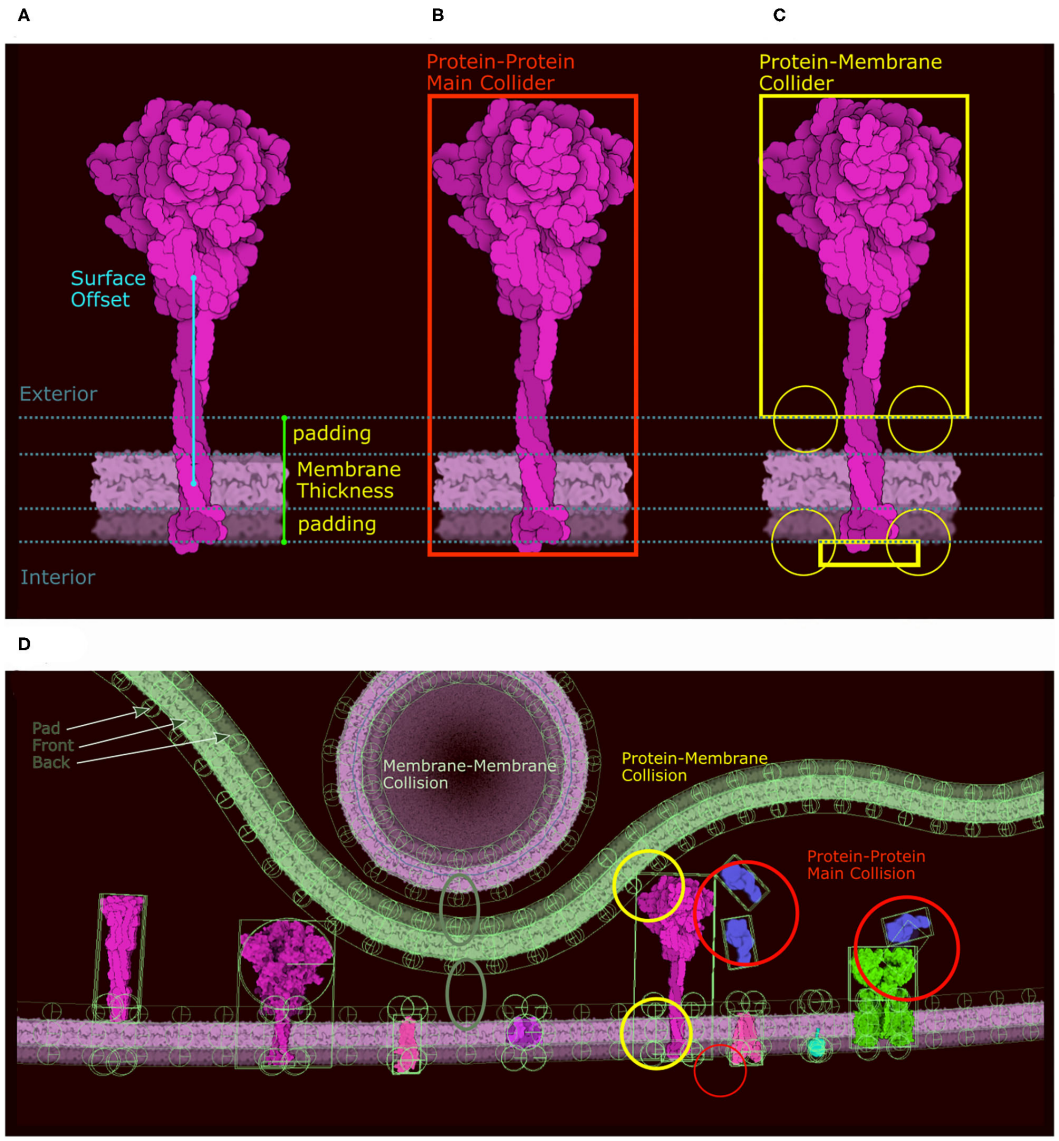
Colliders in CellPAINT. **(A)** Definition of membrane thickness and surface offset (displacement along the Y-axis between the center of the protein and the center of the membrane). **(B)** The *main collider* of a surface ingredient in red. **(C)** The *membrane collider* of the same ingredient in yellow. We define an exterior collider box made of the contour points at the exterior side of the membrane, and an interior collider box made of the contour points at the interior side of the membrane. Two additional circle colliders further anchor the railing system that will constrain the rigid body to remain embedded the membrane. **(D)** Examples of colliders illustrating the different types of collision, as displayed within the Unity editor with protein-protein collision in red, membrane-protein collision in yellow and membrane-membrane collision in green.

Membrane-bound proteins are the most complex ingredients. In the original version, we described our system using a train metaphor, where the membrane is a train track and the membrane protein and its collider are the train and its wheels. In the new version we use the same analogy, but incorporate a more complex collider system to solve stability issues with the initial method. Moreover, different colliders are required for the different collisions that can happen in the scene (protein-membrane, protein-protein, protein-fiber, etc.). As a result, we define two distinct groups of colliders with different roles that are carried out through the physics layer tags. The first group only collides with membranes using a combination of colliders, and the second group only collides with other proteins or fibers using the main collider (in red in Figure 5B). For the first group (membrane collision), we proceed as follows: Given the membrane thickness, a padding value, and the surface offset along the Y axis, we divide the molecule sprite into three clusters of contour points: the exterior side of the membrane, the intramembrane portion that covers the membrane, and the interior side of the membrane (Figure 5A). The intramembrane points are ignored and we used the exterior and interior points, if any, to generate a collider as we do for the main collider using eigen decomposition. In addition to these two shape-based colliders (top and bottom) we create circular colliders on either side of the membrane, two above and two below (in yellow in Figure 5C). These colliders lock the protein on the membrane like two wheels on either side of a rail represented by the membrane collider. Figure 5D summarizes the different types of colliders and the collisions they control. Colliders are defined at the ingredient creation step, either when loading a recipe or when using the “Create Ingredient” tool. The “Create Ingredient” tool allows users to define the relative orientation of the protein and the membrane-spanning portion using two simple sliders, while the recipe is a dictionary that defines the relative position and the sprite image to use. In these definitions of orientations and offsets, CellPAINT assumes that the orientation is always Y-up.

### Refining a Scene With Interactions and Pinning

Biomolecular interaction is the basis of most processes in life, so any cellular modeling tool must necessarily incorporate methods to capture interactions. In many cases, dedicated interaction surfaces on molecular subunits create assemblies with defined geometry: just think of the beautiful symmetry (and quasisymmetry) of icosahedral viruses. Increasingly, we're also seeing examples where intrinsically-disordered chains interact to form functional assemblies or aggregates with structures that are harder to define programmatically.

In CellPAINT, we have taken the first steps toward incorporating these types of interactions into the painting process with two simple tools that provide consistent behavior and are accessible through the turnkey user interface. First, a “Pin-to” tool allows users to click on a local position in two sprites and constrain the distance between those points. This constraint is applied isotropically, so the interaction does not enforce a particular geometry of interaction. Specific interactions are currently an active area of development in the project. Second, a “Pin” tool allows users to freeze ingredients or groups of ingredients in place. When combined with the “Nudge” tool, this allows users to coax and freeze molecules into desired relative conformations.

### Hierarchical Assembly With Grouping and Locking

The ultimate goal in mesoscale work is to approach the modeling of entire cells. Unfortunately, this is not currently feasible with current software. The Unity engine driving CellPAINT encounters unacceptable issues with speed and instability with scenes with ~2,000 soluble ingredients, so dynamic scenes of several hundred nanometers wide, at typical cellular packing densities, are the current limit. Incorporation of molecules with additional constraints (fibers, membrane proteins, pinned molecules, etc.) further limits the complexity of scenes that may be created with interactive performance. We have incorporated two forward-looking tools into CellPAINT-2.0 to test methods for scaling illustrations to much larger scenes. These types of tools, in combination with software-based optimizations, such as Entity Component Systems or hardware-based approaches, such as GPU rigid body physics as implemented in NVIDIA PhysX v4.0, will chart a path toward modeling entire bacterial cells and beyond.

The “Group” tool is designed to simplify the generation of assemblies or repeating elements within scenes, taking advantage of the hierarchical nature of cellular structure. Multiple molecules are chosen in the scene and then grouped together to create a new brush composed of the entire collection. In this way, hierarchical structures may be built and then stamped into the scene to build higher levels of hierarchy. This collection can comprise all types of molecules and also all linked molecules (using the “Pin-to” tool). Within these groups, each instance of the molecule retains its physics, allowing each collection to continue to interact dynamically with its surroundings when brushed into the scene.

**Figure 6:**
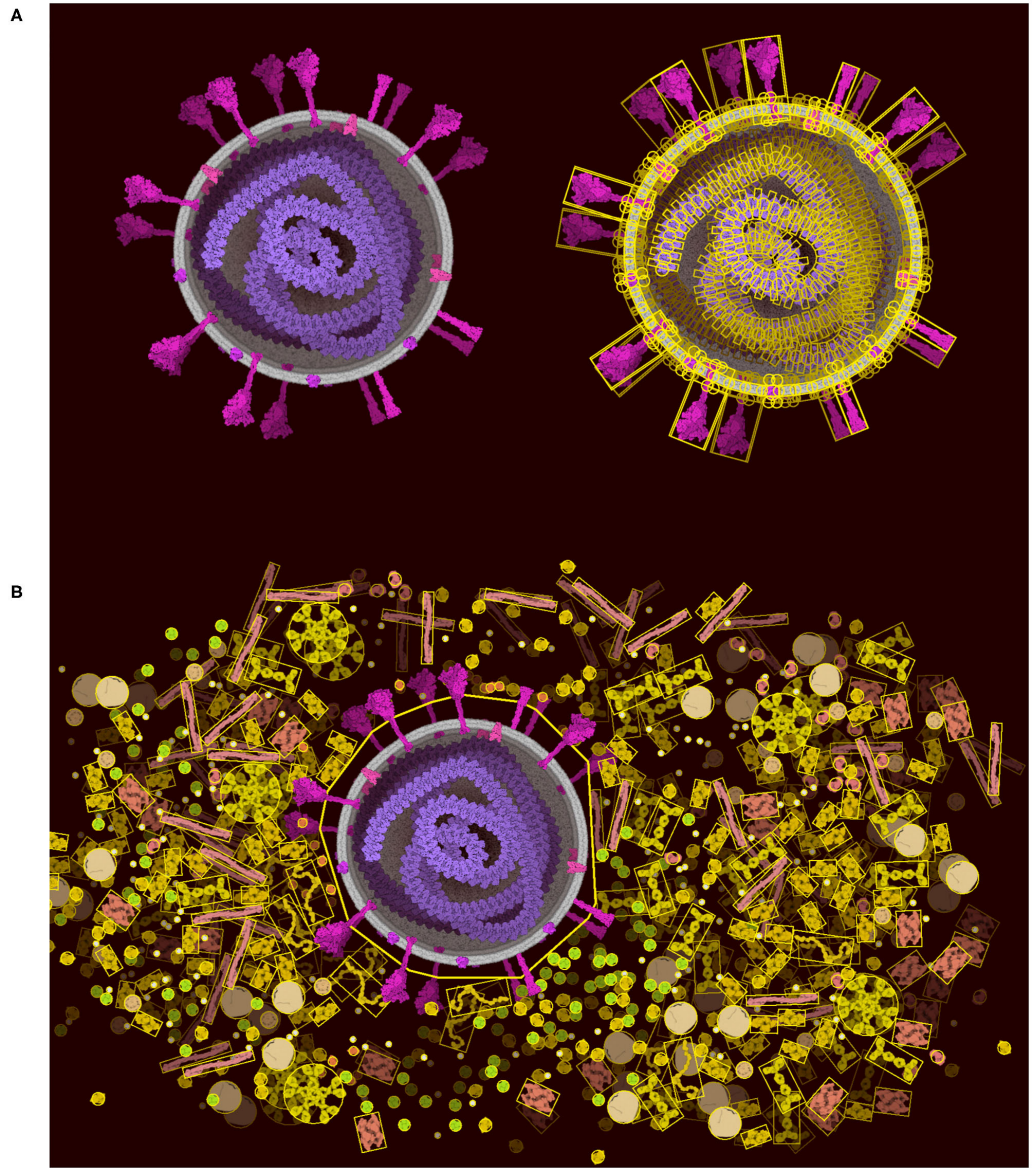
Lock option and collider simplification. **(A)** A coronavirus drawn using the default ingredients in CellPAINT (left), with an overlay that shows all of the colliders used during brushing (right). **(B)** After locking the ingredients in the virus, a simple 2D polygon collider is calculated, which then excludes other ingredients in the scene, such as the surrounding blood plasma proteins shown here.

The “Lock” tool directly addresses the limitations of Unity, allowing users to lock portions of the scene and vastly reduce the number of constraints that must be optimized in the scene. The physics is turned off between all of the components selected in the locked portion (Figure 6A). The portion is then treated as a rigid body represented by larger colliders made of 2D polygons (Figure 6B). The 2D polygons are defined using the Accord.NET library (http://accord-framework.net), using the Graham convex hull that envelopes the N clusters of closest ingredient center positions (8 nm distance cutoff). The clusters are calculated using the mean shift clustering approach. In this way, highly complex scenes may be constructed piece-by-piece, locking portions as they are finished, and brushing additional ingredients around them. As illustrated in Figure 6B we can clearly see the trade-off as we lose the spike collision and add a collision space above the membrane.

We have also used several alternative methods to build large scenes. First, the user can save a screenshot of finished portions of the scene and re-import them as background. The user can then continue to build the scene around the background image, progressively saving and importing the image as regions are filled in. Second, users can create new ingredients, in CellPAINT or with 3rd-party tools, that represent a full virus or even an organelle and import them as large brushes. The only limitation is that these images need to be generated with a transparent background. For both of these options, CellPAINT includes methods to import the images and define the appropriate scale.

### The Elusive Undo Button

Because cellPAINT is dynamic, there is an intrinsic difficulty in creating classic features, such as undo and redo. For example, when the user places an ingredient and turns on diffusion, the ingredient moves a significant distance away from its original placement making its removal during an undo event potentially confusing to the user. In addition, if the ingredient is later added to a group, locked, or pinned to another ingredient, an undo or redo event could introduce instability in the physics simulation. The undo/redo system will be part of our next development round with a focus on performance (e.g., enhancements based on GPU or entity component system).

Currently, the erase tool is the primary functionality to help users correct mistaken actions. We have provided several options for the erase tool to make it as flexible as possible, including erasing of individual misplaced objects or selecting all instances of a particular molecule, allowing the user to start over. We are currently developing several additional enhancements for the erase tool, including masking particular compartments or layers, and defining a radius to the eraser, to allow a “brush erase” of large segments of the scene. Extending the drag tool to allow repositioning of any element in the scene was provided to help the user in case of high number of collisions that could trigger the simulation to stop. Namely, user can displace locked items, groups and large selections. In addition, the save/restore options allow the traditional approach of backing up partial versions of a particular project, allowing users to return to previous versions if fatal errors are made.

### Moving Toward Quantitative Modeling

We are exploring applications of CellPAINT as a tool for research. In particular, we are testing it as a tool for hypothesis generation, allowing researchers to pose “I wonder if…” questions, and then rapidly test them by seeing if they are consistent with the known size and geometry of the molecular players. We have incorporated several tools to streamline these efforts in moving CellPAINT illustration toward a more quantitative approach. A “Measurement” tool allows users to measure (and continually monitor) distances between selected molecules in the scene. A movable scale bar is also provided to monitor the current magnification of the canvas. To help control concentrations as ingredients are added to a scene, the copy number is displayed in the ingredient palette. Finally, in our first step to incorporating experimental information on ultrastructure, users can import custom background images, such as slices from cryoelectron tomograms, and scale them to be consistent with the ingredients being added to a scene.

### Saving, Archiving, and Sharing Ingredients and Scenes

One of our goals for CellPAINT is to provide a number of options to allow additional creativity by users. To this end, we have created a core set of functionalities to allow saving and restoring of scenes created with native ingredients, as well as tools for incorporating user-customized ingredients into the interface and into scenes. Ingredients may be read and saved as .png files with transparent backgrounds, and treated as soluble, membrane-bound, or fiber, as discussed above for ingredients generated from PDB files. All colliders are calculated on the fly, but this could be exposed to the user as an advanced feature in future releases.

CellPAINT includes several default recipes, including ingredients to depict coronavirus, exosome (Jimenez et al., 2019), and the HIV/T-cell/blood recipe distributed with the original version of CellPAINT. Users can load custom recipes using the CellPACK format which can be created manually or using Mesoscope (Autin et al., 2020).

Saving a scene will produce a text file recording colors, position and constraints (Pin, PinTo, Lock, Group) for all ingredients. If the user adds an ingredient from the “Create Ingredient” widget, saving will create a zip archive containing this text file, the new ingredient information, and all the sprites associated with the new ingredient. CellPAINT loads a variety of different files, including a json file (e.g., a recipe which is a dictionary describing all the protein and their properties), a text file (e.g., a file describing color, position, constraints that made a scene), a zip file (e.g., a container of text, png, and json files). Image files should be located in the same folder as the recipe file, or directly in the cellPAINT-dedicated data folder. Note that the web version only works with zip files, to carry all the image files.

### Cryo-Electron Tomography (cryo-ET) Data Acquisition and Reconstruction

CellPAINT was tested for utility in a research setting with manually-selected 2D slices from experimental cryo-electron tomograms. Mouse embryonic fibroblast (MEF) cells with GFP-labeled mitochondria (Wang et al., 2012) were cultured on R ¼ Carbon 200-mesh gold electron microscopy grids (Quantifoil Micro Tools) and plunge frozen in a liquid ethane/propane mixture using a Vitrobot Mark 4 (Thermo Fischer Scientific). Cells with desired mitochondrial morphology were identified using a fluorescence light microscope equipped with a cryogenic stage (Leica). Thin vitrified lamellae were prepared by cryo-focused ion beam (cryo-FIB) milling using an Aquilos dual-beam FIB/SEM instrument (Thermo Fisher Scientific) following an automated cryo-preparation workflow (Buckley et al., 2020). Grids containing lamellae were transferred into a 300 keV Titan Krios microscope (Thermo Fisher Scientific), equipped with a post-column energy filter (Gatan) and a K2 Summit direct electron detector camera (Gatan). Tilt series were acquired using SerialEM software (Mastronarde, 2005) with 2° steps between −60 and +60°. Individual tilts were collected in dose fractionation mode at 10 frames per second, with a pixel size of 3.598 Å, and defocus range of −5.1 to −7.8 μm. The total dose per tilt was 0.9 e/Å^2^, and the total accumulated dose for the tilt series was under 55 e/Å^2^.

Preprocessing of frames was performed in Warp (Tegunov and Cramer, 2019) including motion correction and contrast transfer function (CTF) correction. Alignment of corrected tilt series was performed in IMOD (Kremer et al., 1996) using patch tracking. Tomograms were reconstructed from aligned tilt series with CTF correction using Warp. Tomograms were processed with a deconvolution filter during reconstruction to enhance contrast.

### Mitochondrial, Erythrocyte, and Coronavirus Recipes

Selected ingredients were generated to explore specific hypotheses in the mitochondrial scenes. Membrane-bound proteins include ATP synthase (PDB ID 6cp6), TIM (7cgp), TOM (7ck6), SAM (6wuh), and the ER-bound ribosome-translocon complex (3j7q). Soluble molecules included cytoplasmic ribosomes (4ug0), mitochondrial ribosomes (6gaw), actin filaments (6bno), and the mitochondrial intermembrane protein cytochrome c (3cyt). In some cases, orientations chosen automatically by CellPAINT did not correspond to the hypotheses, so sprites were generated in Mesoscope and imported into CellPAINT. Similarly, the folded conformations of MFN1/MFN2 were generated by homology modeling using I-TASSER (Yang et al., 2015) based on PDB entries 2j69, 2j68, 5gof, 6jfk, and 2w6d, rendered in Mesoscope and imported. Extended conformation of MFN2 was generated by homology modeling using SWISS-MODEL (Waterhouse et al., 2018) based on template PDB 2w6d.

Ingredients for the distinctive erythrocyte membrane cytoskeleton were based on several reviews (Baines, 2010; Lux, 2016). The large protein complexes are based on a modeling study (Burton and Bruce, 2011), and were built manually using the Python Molecule Viewer (Sanner, 1999) and rendered in Illustrate. Several ingredients were modeled, rendered in Illustrate, and then imported into CellPAINT. Band 3 complex included Band 3 protein (4yzf and 1hyn), glucose transporter GLUT1 (4pyp), and glycophorin A (1afo). The macrocomplex was built around the Band 3 complex, adding protein 4.2 (based on 4pyg), RHAG (3hd6), ankyrin (4rlv and 4d8o), ICAM (4oia), and CD47 (5tzu). The junctional complex was modeled from the Band 3 complex with additional glycophorins (1afo), protein 4.1 (3qij), protein 4.2, and p55 (4wsi). An actin protofilament was also built around the actin-tropomyosin complex in PDB entry 2w4u, adding tropomodulin (4pkg, 4pki). The protofilament was pinned to the junctional complex while creating the image in CellPAINT. Spectrin was created from the structure of two alpha chain repeats (1u5p) and imported as a fiber. Two parallel spectrin fibers were drawn, and the ends were pinned to the actin protofilaments and the center was pinned to the macrocomplex.

The interior of the erythrocyte is filled with hemoglobin, modeling from PDB entry 2hhb. Many proteins involved in energy metabolism and antioxidant activity are found in the erythrocyte, and are represented simply here by a generic protein. Blood plasma is illustrated using the default palette provided by default and developed for the earlier version of CellPAINT (Gardner et al., 2018).

The budding coronavirus image was based on previous illustrative work (Goodsell et al., 2020b), and incorporates a number of simplifications using a limited palette of ingredients. The coronavirus ingredients (“Coronavirus” palette) are based on the SARS-CoV-2 v.20-06 mesoscale model (Nguyen et al., 2021) Cytoplasmic ingredients use the default palette developed for T-cell cytoplasm in CellPAINT (Gardner et al., 2018).

## Results and Discussion

### Iterative Development With Beta Testing

Our goal with CellPAINT is to create a tool that is intuitive and easy to use for users who may not be familiar with structural biology. We have followed two overarching design principles to achieve this goal: minimizing the number of tunable parameters so the painting process will remain stable, and providing readily-accessible help to get novice users started.

CellPAINT-2.0 builds on three previous releases. The first version of CellPAINT focused on a scene in HIV biology and was the test bed for the basic approach. Two application-focused versions followed and were used in targeted beta-testing scenarios. *CellPAINT-exosome* was created as part of a summer internship and prototyped the incorporation of hand-drawn illustrations for use as sprites (Jimenez et al., 2019). This laid the groundwork for the “Create Ingredient” widget in CellPAINT-2.0, and also resulted in refinement of the way sprites are handled within the three-layer definition of the scene and in interactions with membranes. More recently, *CellPAINT-coronavirus* was created early in 2020, as a tool to help users explore the science behind the COVID-19 pandemic. A contest was launched at the RCSB Protein Data Bank (Figure 1B), and users were asked to provide direct feedback on usability and a wish list for feature development as part of the contest entry. This feedback led to development and refinement of many of the features in CellPAINT-2.0, which was released in late 2020 and announced on social media. Two features were by far the most requested: an Undo button (described in more detail in a section below) and the ability to add custom ingredients.

As might be expected when dealing with the complexity of biology, the new “Create Ingredient” turned out to be one of the most complicated features to streamline, and many cycles of design and testing were needed to create a tool that was flexible enough but still prevented users from getting lost or stymied. For example, to manage the diversity of PDB files, we added boxes to specify the desired protein assembly (Biological Unit or Asymmetric Unit), specific models when adding NMR structures, and the nomenclature used for chain selection. However, these boxes are provided as optional, and the primary biological assembly, as denoted in the PDB archive, is chosen by default. Dealing with membrane ingredients was also challenging: we had to provide a way to control ingredient rotation and membrane offsets because, in most cases, simply fetching PDB codes was not enough to ensure the correct orientation relative to the membrane. For simplicity, this rotation and offset are provided as two intuitive sliders in the interface, rather than the more comprehensive translation and rotation parameters used in Mesoscope and our other expert tools.

User testing and feedback was also essential for refining the many aspects of user interaction and navigation within the interface. The desire to create “themed” biological scenes inspired the addition of customizable compartments (for example, “cytoplasm,” “blood plasma,” “coronavirus”), that could be populated entirely with custom ingredients and exported as zip files. To complement this, the palette view was modified to facilitate the navigation through compartments, and ingredients can be displayed as a list or as icons, with the option to search ingredients by name with an integrated search bar or remove ingredients directly from the palette. Constructing scenes on top of micrographs required optimization of the tools dedicated to managing multiple background images, so that they now can be rotated, overlaid, and combined with user-definable opacity.

Additional design choices were guided by common problems encountered by users during creation of scenes. For instance, membrane drawing was initially treated as completely free, but after seeing many spuriously-intersecting membranes in user illustrations, we implemented strong constraints to enforce the continuity of membranes as they are drawn. Similarly, fiber drawing is now constrained to avoid intersection with membranes, since some beta-testers were drawing fibers too fast, leading to segments split between the inside and outside of compartments.

The major hurdle in all of these enhancements has been finding the appropriate level of control, given the many tunable parameters that are required to specify CellPAINT sprites and their behaviors. Beta testing has been essential for finding the sweet spot in design of the parameters that are exposed to the user. In particular, the interface for generating custom fibers and membrane-bound proteins posed many challenges in this respect. During design of CellPAINT, we found that the details of colliders are critically important and small changes in parameters can lead to fibers with unstable dynamics or proteins that are frozen in one place in the membrane or are rapidly expelled from the membrane. We ultimately chose a high-level approach, with very few user-tunable parameters, to ensure that in most cases, the user will create an ingredient with stable behavior in the context of other ingredients. As we continue to expand our community of users, we will closely monitor this level of control to ensure that it is not too limiting as users apply it to an increasingly diverse body of applications.

### CellPAINT as a Tool for Hypothesis Generation

CellPAINT is designed to have a tight connection to experimental data from structural biology. As such, we are exploring application of CellPAINT as a tool for hypothesis generation. The goal is to allow researchers to ask “what if” questions about the molecular details of cellular ultrastructure, and then answer these questions interactively by building a cellular scene with appropriately-sized and interacting components. As a first step toward this goal we tested application of CellPAINT in the interpretation of 2D slices from cryoelectron tomograms.

#### Interpreting Mitochondrial Tomograms

**Figure 7:**
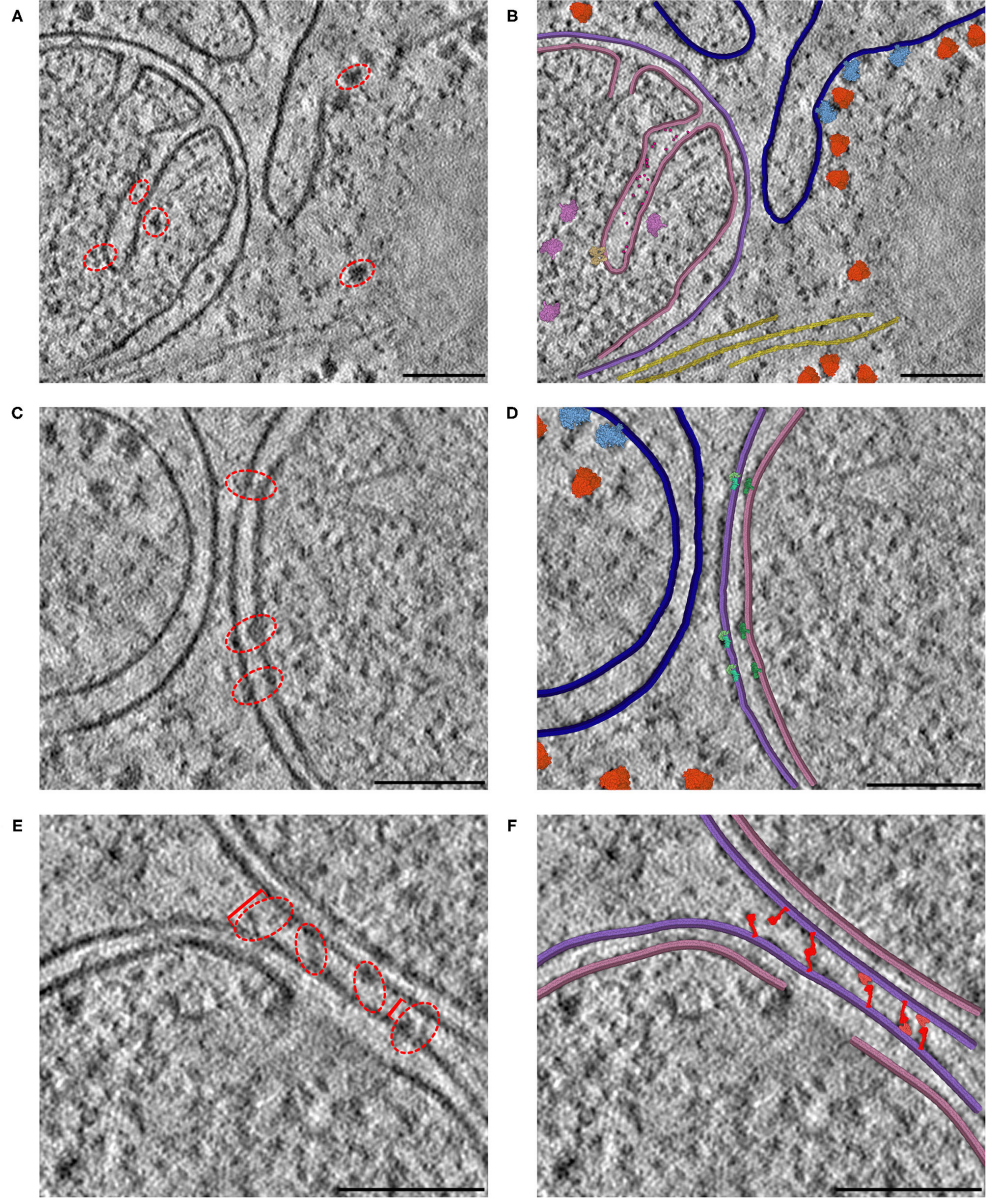
Interactive hypothesis testing with cryo-ET sections. **(A)** Mitochondrion with cristae, endoplasmic reticulum and actin bundle. Densities circled in red are examples of densities interpreted using CellPAINT. **(B)** Interpretation with mitochondrial outer membrane in purple, inner membrane in pink and ER in navy blue. ATP synthase in tan, mitochondrial ribosome in plum, cytochrome c in hot pink, soluble cytoplasmic ribosomes in orange, membrane-bound ribosomes in light blue, and actin in yellow. **(C)** Mitochondrion adjacent to endoplasmic reticulum. **(D)** Interpretation with SAM50 complex in light green, TOM40 complex in aquamarine, and TIM22 complex in dark green. **(E)** Mitochondrion-mitochondrion interface. Brackets in red indicate measured distances between OMMs of the observed mitochondrial interface, where the furthest distance is 34 nm (left bracket) and closest distance is 18 nm (right bracket). **(F)** Interpretation with MFN1/2 “extended” in red and “folded” in salmon. The break in the lower membrane is an example of a region where the membrane is not perpendicular to the plane of the slice. Scale bars 100 nm.

The resolution of cryoelectron tomography is rapidly approaching the level of individual proteins. In many cases, however, only large complexes like ribosomes, cytoskeletal elements and membranes are easily recognizable. We are using CellPAINT as a preview of what an interactive tomography-interpretation tool might look like. In the current version, it is simple to paint in membranes and fill them with proteins, both those that can be seen in the tomogram and those that we know are there from proteomics but can't quite resolve. Figure 7 includes three distinct 2D slices from a single tomogram of a cryo-FIB-milled lamella of a mouse embryonic fibroblast (MEF) cell. In all three slices, we used CellPAINT to model both the outer mitochondrial membrane (OMM) and the inner mitochondrial membrane (IMM) (Figure 7A). The IMM forms highly-curved functional folds that harbor many protein complexes involved in both energy production and the activation of cell fate pathways, such as apoptosis. To further contextualize these functional compartments, we placed structures of proteins involved in both of these processes, including ATP synthase dimers and cytochrome c, in regions that contained matching densities visible within the tomogram (Figures 7A,B). In addition to mitochondrial membranes, we modeled the endoplasmic reticulum (ER) membrane as well as cytoplasmic actin filaments. We also observed densities in our tomogram for three different varieties of ribosomal complexes: cytosolic, ER membrane-bound, and mitochondrial. As structures for each of these distinct ribosome varieties were available in the PDB, we were able to represent and model these as distinct entities within the CellPAINT scene (Figures 7A,B).

We observed several mitochondrial membrane-associated densities that we were unable to unambiguously identify based on previous tomographic characterization (Figures 7C,E, red dashed circle). First, we observed intriguing membrane protruding densities that appeared to form a putative connection between the IMM and OMM in our tomogram (Figure 7C, red dashed circle). We hypothesize that these densities correspond to the translocases of the inner and outer membrane (TIM/TOM) complexes responsible for mediating import of the majority of mitochondrial resident proteins (Wiedemann and Pfanner, 2017) To test this, we used CellPAINT to import and overlay deposited structures of TIM22, TOM40, and SAM50 complexes within our tomogram. We observed substantial overlap in both the overall shape and fit of these maps within these tomographic densities, suggesting that these may correspond to these import channels.

We also observed bridging densities that appeared to connect two mitochondria that were in close proximity to each other. We predict these densities represent mitofusion proteins that function as active tethers forming hetero- or homo-dimers across adjacent mitochondria to facilitate mitochondrial fusion (Li et al., 2019). Interestingly, we noticed that both the OMM-OMM distance and the length of these bridging densities varied along this interface, ranging from 34 nm at the farthest point to 18 nm at the closest point (Figure 7D). We hypothesize that these variations correspond to distinct, GTPase-coupled conformations of MFN1/2 proteins that represent successive stages of the mitochondrial fusion process. There are currently no full-length structures of the functional MFN1/2 proteins, however there are available structures of truncated versions of these proteins (Li et al., 2019). We used homology modeling to generate structures of these complexes with distinct templates to represent predicted conformations of these proteins, in both “extended” and “folded” conformations (Yan et al., 2018). We imported these structures as sprites in our CellPAINT scene and were able to match these structures to densities we observed bridging the mitochondria-mitochondria interface. Consistent with our predicted model, we observed that extended conformations more closely matched densities present at the farthest points (34 nm), whereas a mix of extended-closed conformations matched densities present at the more closely appressed OMM-OMM regions.

#### Current Limitations of CellPAINT in Hypothesis Generation

Using CellPAINT, we assigned several structures to distinct tomographic densities within our data to generate models of mitochondrial membrane architecture. Furthermore, we used CellPAINT as an interactive tool to test hypotheses regarding the identity of unknown tomographic densities by overlaying different predicted protein structures and asking whether the overall shape and spatial constraints matched those present within our experimental tomographic data. We anticipate that CellPAINT will be a useful predictive tool upstream of tomographic structure-solving techniques, such as subtomogram averaging to determine whether putative densities present within tomograms represent desired protein structures. Overall, this tool allowed us to quickly, easily, and interactively interpret our cellular tomograms by directly interfacing with the wealth of *a priori* protein structure information present within the Protein Data Bank.

However, there were several limitations that kept us from fully utilizing CellPAINT as a tool for hypothesis generation in this context. We found that it was difficult to interactively adjust certain orientation parameters of the model to accurately overlay ingredients onto the tomographic densities. As a workaround, we used Mesoscope to generate sprites with multiple orientations and imported them into cellPAINT. Most importantly, CellPAINT is currently limited to interpreting a single, two-dimensional slice of the tomographic data. Although much information can be captured in a single view (as demonstrated in Figure 7), for complex three-dimensional cellular microenvironments, such as mitochondrial cristae, it is often difficult to select a single 2D slice that encompasses all desired complexes. In some regions we were forced to represent truncated portions of the membrane to match the tomographic densities (for example, the discontinuous IMM in Figures 7B,F), even though 3D views representing other 2D slices show clear continuous membrane structures. We will use insights gained in this study to guide development of the 3D version of CellPAINT, with the goal of allowing this type of hypothesis testing in 3D spaces.

### Moving Toward the Cellular Mesoscale

**Figure 8:**
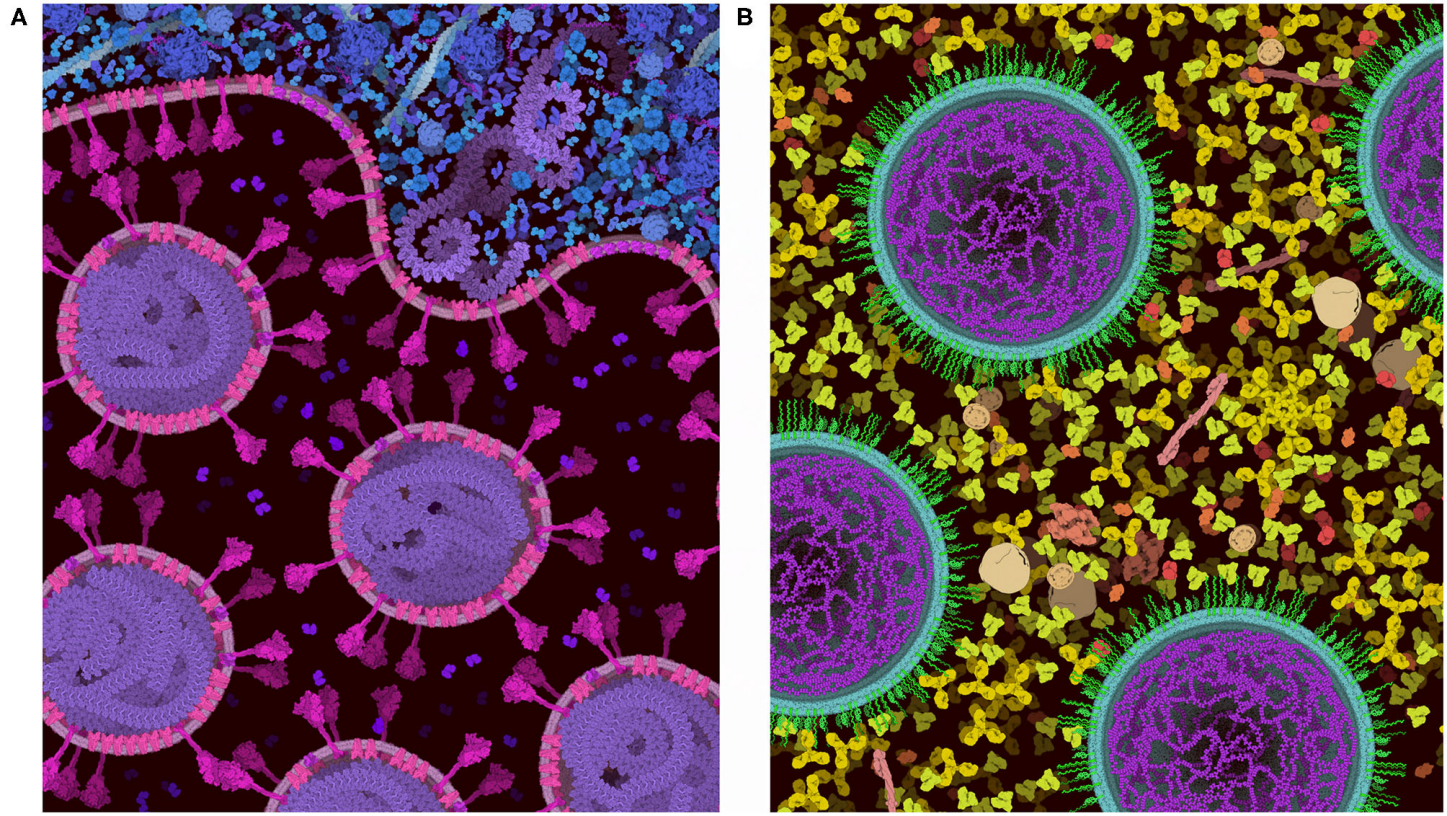
Images of coronavirus biology created using “Group” and “Lock.” **(A)** Coronavirus particles budding into an endosome, with cytoplasm at top in blue. **(B)** Idealized conception of coronavirus mRNA vaccine, with spike-coding mRNA in purple, and pegylated lipid in green, surrounded by blood plasma. The process of creating these two images is described in the text.

Figure 8 includes two examples of using the “Group” and “Lock” functions to build progressively complex images. In both images, a single virus or vaccine particle was constructed first, and then all components were grouped to form a brush. Multiple copies were then added one-by-one, using the nudge tool to make small changes and then locking the entire particle. Finally, background molecules were drawn around the locked particles to complete the scene. For the budding illustration in Figure 8A, the high density of molecules in the cytoplasm was achieved by adding an additional step, where the scene was saved as snapshot and then read in as a background image, and any stray empty spaces caused by the group polygons were filled with molecules. All molecules in these two images are provided in the default palettes except for the pegylated lipid, which was created from idealized coordinates using the stand-alone version of Illustrate, then read as a sprite into CellPAINT.

**Figure 9:**
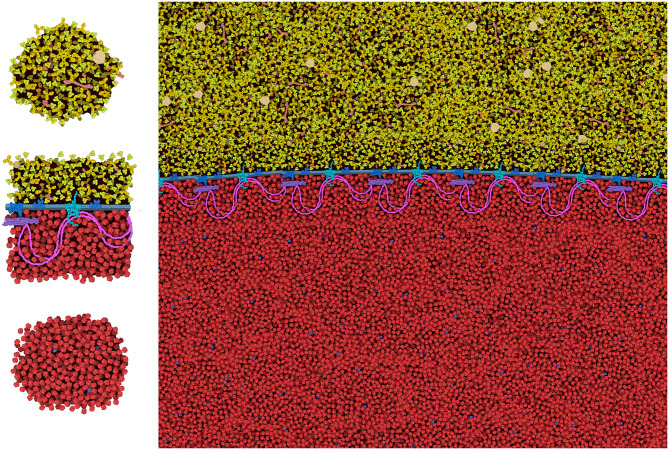
Tiling approach to cellular illustration. Three composite tiles (left) are used as brushes to create an illustration of an erythrocyte cell surface, with hemoglobin at the bottom and blood plasma at the top.

The general features of CellPAINT that allow import of custom sprite images opens the door to experimentation with new methods. For example, we are currently exploring methods to create mesoscale “tiles” that allow the construction of much larger scenes. A simple prototype is shown in Figure 9. With this approach, CellPAINT is used to create a small patch of ingredients, such as a patch of cytoplasm or a segment of membrane filled with proteins, and these patches are used to create composite brushes, allowing rapid creation of large scenes. An early experiment with illustrating an erythrocyte worked surprisingly well with the current tools of CellPAINT. A segment of membrane with one repeat of the spectrin network was generated, saved as an image, and then cut out in Photoshop with a transparent surrounding. This was treated as a membrane-bound protein when imported into CellPAINT, so that we could use the native membrane behaviors of CellPAINT to design the shape of the cell surface and align copies of the membrane patch along it. Two additional brushes were created for patches of blood plasma and hemoglobin-filled cytoplasm. Since these were treated as soluble ingredients, they are given random orientations each time they are painted into the scene, reducing problems with visual periodicity in the final illustration.

Several aspects of the design of these mesoscale tiles proved essential for creating a coherent image. First, the black background needed to be retained in the tile. In this way, when the tiles, the foremost one blocks tiles behind, so the molecules depicted in overlapped tiles don't build increasingly higher concentrations. Still to be resolved are the best approaches for treating molecules at the edges of the tiles. In the figure, we generated tiles with a few floating foreground molecules surrounding the edge. This helps to make the edges less apparent in the final scene, but occasionally causes artifacts, such as the linear strings of visually-higher concentration that are seen just above and below the membrane in the figure. Further experimentation will be needed to reduce these types of artifacts, and also to approach scenes with more complex ultrastructure (think: the complex membrane geometry in chloroplast grana, mitochondrial cristae, or the cellular endomembrane system).

### Modeling Biological Complexity

CellPAINT is part of a larger effort to model the cellular mesoscale, and we have been exploring a variety of approaches, each with advantages and limitations. Traditional illustration (as in Figure 1A) is by far the most flexible approach—the scenes are limited only by imagination and drafting skill. Anything can interact with anything, in any way that we like. These illustrations, however, are inherently qualitative, and great care must be employed to correctly integrate the available body of knowledge into the final image. Programmatic 3D modeling tools, such as our CellPACK suite and Integrative Modeling Platform (Russel et al., 2012) are at the other end of the spectrum: they are designed to be quantitative and adhere closely to the known structures, interactions and behaviors of the ingredients and compartments. These methods are typically used in modes with very little interactive input, and the higher-order structures emerge from properties of the components. Both of these approaches, unfortunately, are largely the domain of experts since they have a steep learning curve for usage.

CellPAINT is designed to work in the domain between these extremes, to make mesoscale modeling accessible to a wider range of users. We feel that perhaps the most important element of this work is the design and testing of approximations that allow turnkey interactive construction, while still capturing enough of the relevant properties to yield a semi-quantitative model of the biology. Within this approach, users in educational settings can design a composition that tells a functional story, while relying on the program to ensure that everything is scaled correctly and with the proper behaviors. Similarly, researchers can pose questions while interpreting their data, again relying on the program to manage the details of the structure and interaction of ingredients, allowing them to focus attention on the biology. The challenge has been to design a level of interactivity and tunability that fulfills these goals.

In our testing, CellPAINT is effective in a central class of biological systems: cellular or viral scenes with simple compartments, more-or-less rigid ingredients, and simple interactions. This allows ready access to illustrations of viral structure, modeling of portions of organelles, such as mitochondria and chloroplasts, secretory vesicles, and the like. The choices made in this version make other types of systems difficult to approach. Systems that rely on intrinsically-disordered proteins are currently difficult to realize in CellPAINT, but may be possible with a more detailed interface for approaching articulation and interaction of ingredients. As described above, size and complexity of systems is also a challenge that we are working to address. The 2.5D paradigm also imposes strong orientational constraints on how objects are placed, so some experimentation must be employed to create scenes that are relevant to the actual biology.

### Advantages and Disadvantages of the 2.5D Paradigm

The 2.5D paradigm employed in CellPAINT is a trade-off between several advantages and disadvantages. All of the ingredients are seen from the same viewpoint, so for example, antibodies always have the iconic Y-shape. This *diagrammatic and easily interpretable view* helps viewers recognize molecules within the scene, and also provides a very direct interactive painting experience. However, the 2.5D metaphor can introduce artifacts in the packing, since these iconic views often produce the largest footprint of the molecule in the plane of the canvas. Similarly, the 3-layer approach imposes non-realistic limitations on the packing that make for easy painting but reduced scientific accuracy. For example, in the 2.5D implementation, fibers are drawn mostly in a single layer or with periodic stochastic jumps between layers. This yields an interpretable image but misrepresents the actual random orientations that we might expect in real systems. Similarly, membranes are drawn perpendicular to the picture plane in 2.5D, to give an easily-interpretable cross section. This strongly limits the types of compartments that may be modeled and depicted. These conventions were largely developed and tested in our traditional paintings and allow many types of scenes to be created in spite of the limitations.

**Figure 10:**
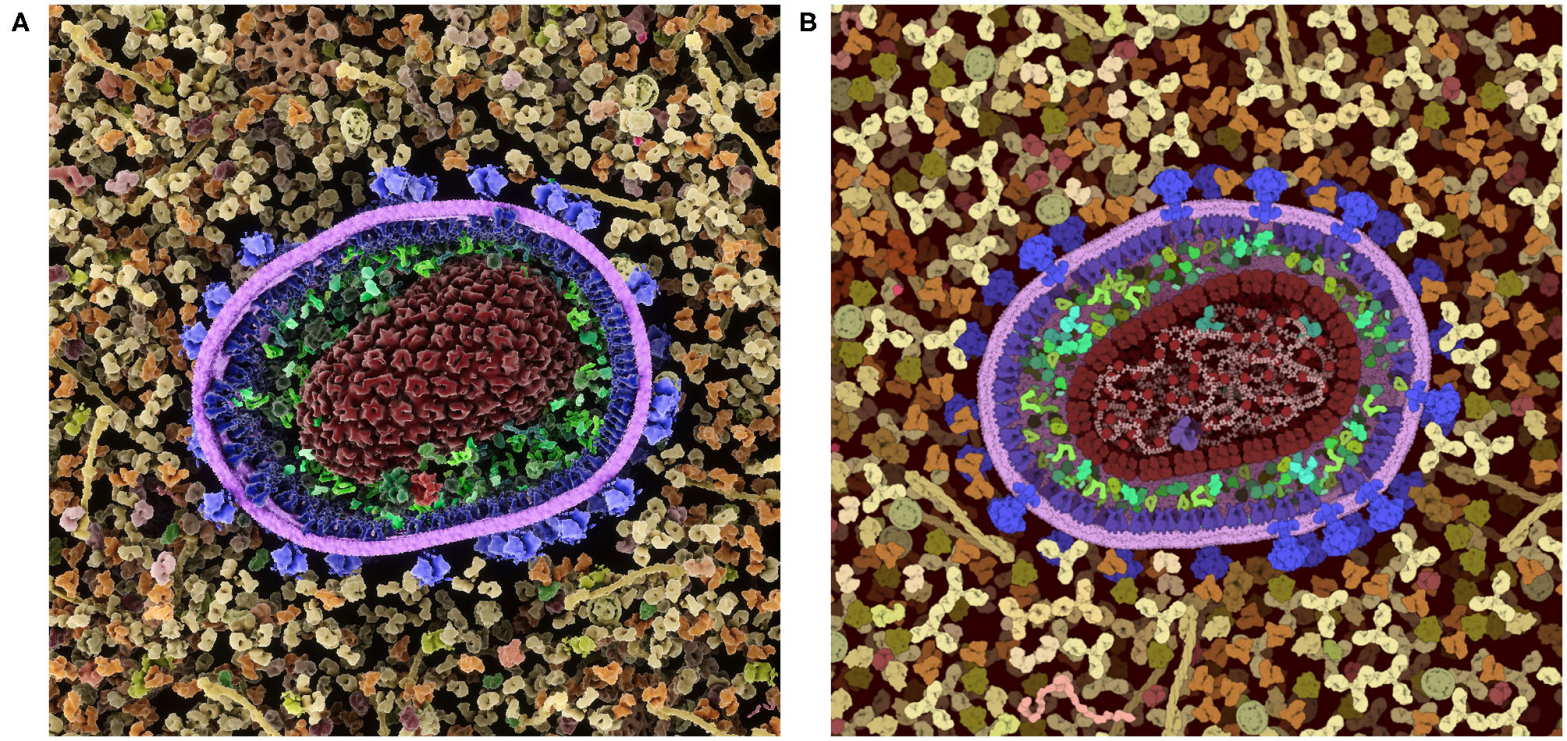
Comparison of a cross-section of HIV-1 in blood plasma created in **(A)** the 3D approach used in CellPAINT-3D and **(B)** the 2.5D approach used in CellPAINT. Each has advantages and disadvantages: for example, the packing of molecules is more physically accurate in 3D, but molecules are easier to recognize in 2.5D (for example, try identifying antibodies in each image).

These inherent benefits and shortcomings in the 2.5D representation have led us to explore a 3D version of CellPAINT in conjunction with a virtual reality version of the program, CellPAINT-VR. The 3D approach promises several useful methodological and application enhancements. In particular, CellPAINT-3D has the advantage of being able to show density and crowding in a more realistic way (Figure 10). For example, objects that aren't spherical or span multiple 2.5D layers pose a challenge to the 2D system that is easily remedied by 3D representations of the ingredients. Higher densities are easier to accomplish in 3D because objects have additional degrees of spatial and rotational freedom to resolve clashes. Also, linear fibers and membranes can be treated more realistically in a 3D paradigm, lifting the restrictions imposed by the 2.5D layers and allowing modeling of membrane-bounded compartments of arbitrary shape and arbitrary orientation of fibers. That being said, the 3D approach is presenting a whole new set of challenges for these challenging molecule types, including turnkey and interpretable methods for clipping and easy methods for defining the location and shape while drawing.

As we continue work on 3D versions, we intend to take advantage of insights we have gained from work in 2.5D. In particular, the layering that is inherent in the 2.5D approach improves the visual interpretability of the scenes. The limitations it imposes in the orientation of membranes and fibers are acceptable in qualitative settings, such as education and outreach, and we have found that the interpretability of 3D scenes can be enhanced by imposing a similar layering approach to the placement of ingredients.

## Conclusions and Future Plans

We have used CellPAINT as a test bed for experimenting with new tools for making the mesoscale more easily accessible. We have also used this opportunity to explore the many features of the user interface that must be designed and tuned to streamline usability. These have included turnkey tools for managing scale relationships and consistent tools for generating new ingredients that anticipate potential problems posed by the vast diversity of biomolecular structures and interactions. With this new version, it is our hope that the new “Create Ingredient” panel will greatly expand the utility of CellPAINT with our community of users.

Development of the method underscored the need for increasingly sophisticated tools, as we expand the capabilities of CellPAINT to encompass larger and more complex scenes, and as we develop 3D versions of CellPAINT. We will continue to explore methods for increasing the performance of the painting methods and underlying physics, to support larger systems. We will also continue to improve the ingredient generation methods. For example, the fiber generation tool will need to be expanded to include controls for persistence length and an interface for defining helical relationships between successive subunits. These types of enhancements pose challenges for the goal of making this a turnkey tool for non-expert users—we don't want users to be faced with 10 sliders to define a helical fiber.

Similarly, more sophisticated approaches to molecular interaction are sorely needed, both for enforcing the interaction as molecules are added to the scene, and for finding turnkey ways for users to define these interactions during ingredient creation. We are also very interested in augmenting the current diffusive simulation with active behaviors in sprites, such as motors and selective channels, which would open the door to all manner of educational applications and back-of-the-envelope research explorations. In addition, all of this work is being performed in the context of the entire CellPACK suite, with the intention of streamlining the interoperation of Mesoscope recipe curation, procedural modeling in CellPACK, and interactive scene generation with the CellPAINT in 2.5D, 3D, and virtual reality.

Most importantly, we also need to continue to focus directly on the needs of our different user communities. The current version is designed for users with little structural biology expertise and has shown success in educational settings. The cryoEM results included in this report represent our first attempt to use CellPAINT as a tool for research. Fortunately, the insights we have garnered about user interaction and biomolecular representation are directly applicable for creation of an expert tool with more features exposed to the user, for use as a tool for mesoscale structural biology research.

## Software Availability

CellPAINT is currently available as a stand-alone version at https://sourceforge.net/projects/cell-paint/files/cellPAINT2D_2.0_ReleaseCandidates. A web-based version, documentation, and tutorials are available at https://ccsb.scripps.edu/cellpaint.

## Data Availability Statement

Publicly available datasets were analyzed in this study. The reconstructed tomogram is available at: doi: 10.5281/zenodo.4606923.

## Author Contributions

AG and LA: conceptualization, methodology, software, validation, data curation, writing original draft, writing review and editing, and visualization. DF and MMa: investigation, methodology, data curation, writing original draft, and visualization. BB and MMe: investigation, methodology, data curation, and writing original draft. AO: supervision, project administration, funding acquisition, and writing review and editing. DAG: conceptualization, methodology, supervision, writing original draft, and writing review and editing. DSG: conceptualization, methodology, validation, data curation, writing original draft, writing review and editing, visualization, supervision, project administration, and funding acquisition. All authors contributed to the article and approved the submitted version.

## Conflict of Interest

The authors declare that the research was conducted in the absence of any commercial or financial relationships that could be construed as a potential conflict of interest.
